# Augmented Anti-Bactericidal Permeability-Increasing Protein Antibody Levels in Rheumatoid Arthritis Patients Complicated by Usual Interstitial Pneumonia

**DOI:** 10.3390/jcm15145433

**Published:** 2026-07-10

**Authors:** Shomi Oka, Takashi Higuchi, Kota Shimada, Misuzu Fujimori, Atsushi Hashimoto, Akiko Komiya, Koichiro Saisho, Norie Yoshikawa, Michita Suzuki, Toshihiro Matsui, Naoshi Fukui, Kiyoshi Migita, Shigeto Tohma, Kenji Itoh, Hiroshi Furukawa

**Affiliations:** 1 Department of Rheumatology, NHO Tokyo National Hospital, 3-1-1 Takeoka, Kiyose 204-8585, Japan; oka-tkb@umin.org (S.O.); takashi.qef@ac.auone-net.jp (T.H.); shige1010ma@outlook.jp (S.T.); kenji-tky@umin.ac.jp (K.I.); 2 Clinical Research Center for Allergy and Rheumatology, NHO Sagamihara National Hospital, 18-1 Sakuradai, Minami-ku, Sagamihara 252-0392, Japan; komiya.akiko.zy@mail.hosp.go.jp (A.K.); ninja02matsui@gmail.com (T.M.); n-fukui@idaten.c.u-tokyo.ac.jp (N.F.); 3 Department of Rheumatology, NHO Sagamihara National Hospital, 18-1 Sakuradai, Minami-ku, Sagamihara 252-0392, Japan; kouta_shimada@tmhp.jp (K.S.); hako@happy.email.ne.jp (A.H.); 4 Department of Rheumatic Diseases, Tokyo Metropolitan Tama Medical Center, 2-8-29 Musashi-dai, Fuchu 183-8524, Japan; 5 Department of Rheumatology, NHO Himeji Medical Center, 68 Hon-machi, Himeji 670-8520, Japan; misuzufujimori@yahoo.co.jp; 6 Department of Internal Medicine, Sagami Seikyou Ganka Naika, 6-2-11 Sagamiohno, Minami-ku, Sagamihara 252-0303, Japan; 7 Department of Clinical Laboratory, NHO Sagamihara National Hospital, 18-1 Sakuradai, Minami-ku, Sagamihara 252-0392, Japan; 8 Department of Orthopedics/Rheumatology, NHO Miyakonojo Medical Center, 5033-1 Iwayoshi-cho, Miyakonojo 885-0014, Japan; saisho889@miyazaki-catv.ne.jp (K.S.); nori1004@me.com (N.Y.); 9 Tanimura Hospital, 10-2 Kitakoji, Nobeoka 882-0041, Japan; 10 Department of Internal Medicine, NHO Nagoya Medical Center, 4-1-1 Sannomaru, Naka-ku, Nagoya 460-0001, Japan; suzuki.michita.cb@mail.hosp.go.jp; 11 Department of Life Sciences, Graduate School of Arts and Sciences, The University of Tokyo, 3-8-1 Komaba, Meguro-ku, Tokyo 153-8902, Japan; 12 Clinical Research Center, NHO Nagasaki Medical Center, 2-1001-1 Kubara, Omura 856-8562, Japan; migita@fmu.ac.jp; 13 Department of Gastroenterology and Rheumatology, Fukushima Medical University School of Medicine, 1 Hikarigaoka, Fukushima 960-1295, Japan; 14 Department of Internal Medicine, St. Francis Hospital, 9-20 Komine-machi, Nagasaki 852-8125, Japan

**Keywords:** rheumatoid arthritis, anti-bactericidal permeability-increasing protein, antibodies, usual interstitial pneumonia

## Abstract

**Objective:** Chronic lung diseases (CLDs), for example, interstitial lung disease, manifest as an extra-articular complication of rheumatoid arthritis (RA). The contribution of anti-bactericidal permeability-increasing protein antibodies (BPI Abs) in lung involvement in RA or primary Sjögren’s syndrome has been reported. Studies related to anti-BPI Abs in RA with CLD are infrequent. Here, the involvement of anti-BPI Abs with RA and CLD complications was evaluated. **Methods:** Enzyme-linked immunosorbent assays were used to measure anti-BPI Abs in RA sera. **Results:** Higher anti-BPI Ab amounts were present in the RA with usual interstitial pneumonia (UIP) than without CLD (mean ± standard deviation, 10.4 ± 23.5 [ng/mL] vs. 1.3 ± 3.8, *p* = 0.0020). Area under the curve values of receiver operating characteristic curves for anti-BPI Ab and Krebs von den lungen-6 were alike between RA with UIP and without CLD (0.8616, 95% CI 0.8184–0.9048; 0.8716, 95% CI 0.8151–0.9282, *p* = 0.5933, respectively). A relationship between anti-BPI Ab and anti-carbamylated protein Ab levels was observed in RA patients (rho 0.3508, *p* = 1.47 × 10^−14^). **Conclusions:** Anti-BPI Abs were related to UIP in RA patients and might be biomarkers for UIP. These findings predict anti-BPI Abs involvement in UIP pathogenesis in RA.

## 1. Introduction

Examples of chronic lung diseases (CLDs) commonly linked to rheumatoid arthritis (RA) are airway disease (AD), emphysema and interstitial lung disease (ILD), which grant a poor prognosis to RA patients [1,2,3,4,5], and this is worsened by usual interstitial pneumonia (UIP) [6]. Thus, the pathogenic role of CLD in RA requires clarification.

Biomarkers for idiopathic pulmonary fibrosis, including Krebs von den lungen-6 (KL-6) and surfactant protein-D (SP-D), might be applicable for ILD in RA [7,8]. The cut-off levels of KL-6 and SP-D were set for idiopathic pulmonary fibrosis and ILD in collagen diseases and were higher for their application to RA-ILD. Thus, the sensitivities are not sufficient [9]. Antibodies (Abs) to immunoglobulin (Ig) G Fc regions, termed rheumatoid factors (RFs), are related to ILD in RA [10,11]. Posttranslational modifications of lysine residues are involved in generating anti-carbamylated protein (CarP) Abs against homo-citrullinated peptides, which are linked to ILD in RA [12,13]. Thus, some biomarkers are potentially useful for ILD in RA [14]. Because radiological exposure could not be ignored during the examination of chest computed tomography, diagnostic biomarkers would be useful to select RA patients to be examined for chest computed tomography. If these biomarkers could be used as prognostics, severity, development or progression, or treatment-response biomarkers in the future, it will be clinically useful.

Bactericidal permeability-increasing protein (BPI), a bactericidal factor for Gram-negative bacteria, neutralizes endotoxins, is present in neutrophil azurophil granules, and is targeted by anti-neutrophil cytoplasmic Abs (ANCAs). These anti-BPI Abs were detected in systemic vasculitises, cystic fibrosis, RA, and inflammatory bowel diseases [15,16,17,18,19,20]. Anti-BPI Abs are associated with decreased pulmonary function of cystic fibrosis patients, leading to a poor prognosis [21,22]. Anti-BPI Abs are also linked to lung involvement in RA or primary Sjögren’s syndrome [23,24]. Furthermore, ANCAs are involved in ILD in RA [25]. However, the contribution to RA with CLD by anti-BPI Abs is poorly understood and it might have the potential to be a better biomarker for CLD, especially UIP, in RA. The relevance of anti-BPI Abs in CLD, especially UIP, complicated by RA, was examined.

## 2. Materials and Methods

### 2.1. Patients

It was a retrospective study and 657 RA patients with chest computed tomography were recruited at hospitals organized by the NHO Tokyo National Hospital and 55 healthy controls (HCs) were also enrolled in this study. All RA patients satisfied Rheumatoid Arthritis Classification Criteria [26] or American College of Rheumatology criteria for RA [27]. RA patients with a history of exposure to dust or thoracic irradiation for cancer therapy were excluded from this study. Those RA patients were examined by chest computed tomography to establish diagnoses of AD, UIP, emphysema, nonspecific interstitial pneumonia (NSIP), or no CLD as previously described [28,29,30]. CLD was diagnosed without overlap on the basis of the most predominant findings of chest computed tomography images. ILD comprised patients with UIP or NSIP, and CLD(+) included those with NSIP, emphysema, UIP, or AD. Supplementary Table S1 shows the clinical traits of RA patients. Whole blood was obtained from peripheral venous blood from the participants, serum samples were separated after centrifugation at 1500× *g* for 10 min, and stored at −80 °C before antibody detection.

Our study was approved and reviewed by the Research Ethics Committees of NHO Tokyo National Hospital (190010, Approval Date: 29 May 2019) and additional organizations in this research project. Informed consent (written) was given by the patients in this study, performed in accordance with the tenets of the Helsinki Declaration.

### 2.2. Laboratory Measurements and Clinical Assessment

Measurement of anti-BPI Abs was achieved by enzyme-linked immunosorbent assays (ELISA) (Human anti-BPI-Ab Kit), following the instructions of the manufacturer (MyBioSource, San Diego, CA, USA). Sera were diluted 1:10 using the sample diluent from the kit. RF IgA was evaluated with Rheumatoid Factor IgA kits (Organtech Diagnostika, Mainz, Germany). A cut-off value of 4.6694 for anti-BPI Ab was defined from the 98th percentile of HCs, because the cut-off values of autoantibodies could not be defined using sera from RA patients. RF was measured by an N-latex RF kit (Siemens Healthcare Diagnostics, München, Germany). A human anti-CarP ELISA kit was used to measure anti-CarP Abs (Wuhan Fine Biotech Co., Ltd., Wuhan, China). A Picolumi KL-6 Electrochemiluminescence immunoassay system was used to measure KL-6 (EIDIA Co., Ltd., Tokyo, Japan). An SP-D Yamasa EIA II kit was used to measure SP-D (Yamasa Corporation, Choshi, Japan). RF, RF IgA, SP-D, anti-CarP Ab, and KL-6 measurements in some RA cases were reported prior to the current study [13]. Steinbrocker stages were evaluated according to a previous method [31].

### 2.3. Statistical Analysis

Anti-BPI Ab amounts in RA subsets were compared to RA without CLD (RA−CLD) or HCs by *t*-tests. Anti-BPI Ab positivity was analyzed between RA subsets and RA−CLD using Fisher’s exact test (with 2 × 2 contingency tables). Spearman’s rank correlation coefficient rho values were used to assess correlations between autoantibodies. Multiple logistic regression analyses were used to generate a complex marker and performed to assess the independent association of anti-BPI Ab from other factors. Comparisons between RA with UIP (RA)+UIP and RA−CLD were made on receiver operating characteristic (ROC) curves. Area under curve (AUC) values for ROC curves with 95% confidence intervals (CIs) were used for comparisons to AUC values of additional ROC curves by Chi-square analysis. Estimations of optimized cut-off levels were determined based on the highest Youden index.

## 3. Results

### 3.1. Anti-BPI Abs Are Markedly Higher in RA+UIP than RA−CLD

**Figure 1:**
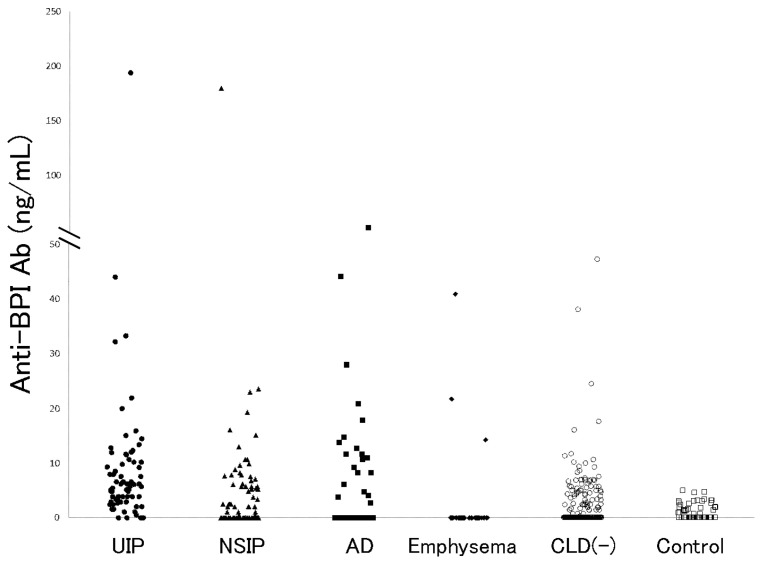
**Evaluation of anti-BPI Ab levels in RA patients and controls.** Distribution of anti-BPI Ab levels is shown. Filled circles, filled triangles, filled squares, filled diamonds, empty circles, and empty squares represent RA+UIP, RA with NSIP, RA with AD, RA with emphysema, RA without CLD, and controls, respectively. RA: rheumatoid arthritis, UIP: usual interstitial pneumonia, NSIP: nonspecific interstitial pneumonia, AD: airway disease, CLD: chronic lung disease, BPI: bactericidal permeability increasing protein, Ab: antibody.

Figure 1 and Table 1 show that anti-BPI Ab amounts were markedly greater in RA+UIP compared to RA−CLD (mean ± standard deviation [SD], 10.4 ± 23.5 [ng/mL] vs. 1.3 ± 3.8, *p* = 0.0020). Anti-BPI Ab amounts were also greater in RA with ILD (7.2 ± 20.9, *p* = 0.0004) and CLD (4.2 ± 15.1, *p* = 0.0004). Anti-BPI Ab positivity was markedly higher in RA+UIP than RA−CLD (n, 44 [62.9%] vs. 42 [14.8%], *p* = 4.63 × 10^−15^) (Table 2). This was also seen for RA with ILD (73 [43.7%], *p* = 3.23 × 10^−11^), NSIP (29 [29.9%], *p* = 0.0015), and CLD (93 [24.9%], *p* = 0.0017). This indicated anti-BPI Abs were linked to RA+UIP. To eliminate the effects of the clinical characteristics of RA patients, multiple logistic regression analyses were also performed (Supplementary Table S2). An association of UIP with anti-BPI Ab remained when conditioned on the clinical characteristics, suggesting that anti-BPI Ab was independently associated with UIP. Multiple logistic regression analyses of anti-BPI Ab were also performed to elucidate the independent association of anti-BPI Ab from KL-6 or SP-D. An association of UIP with anti-BPI Ab remained when conditioned on KL-6 or SP-D, suggesting that anti-BPI Ab was independently associated with UIP (Supplementary Table S3).

**Figure 2:**
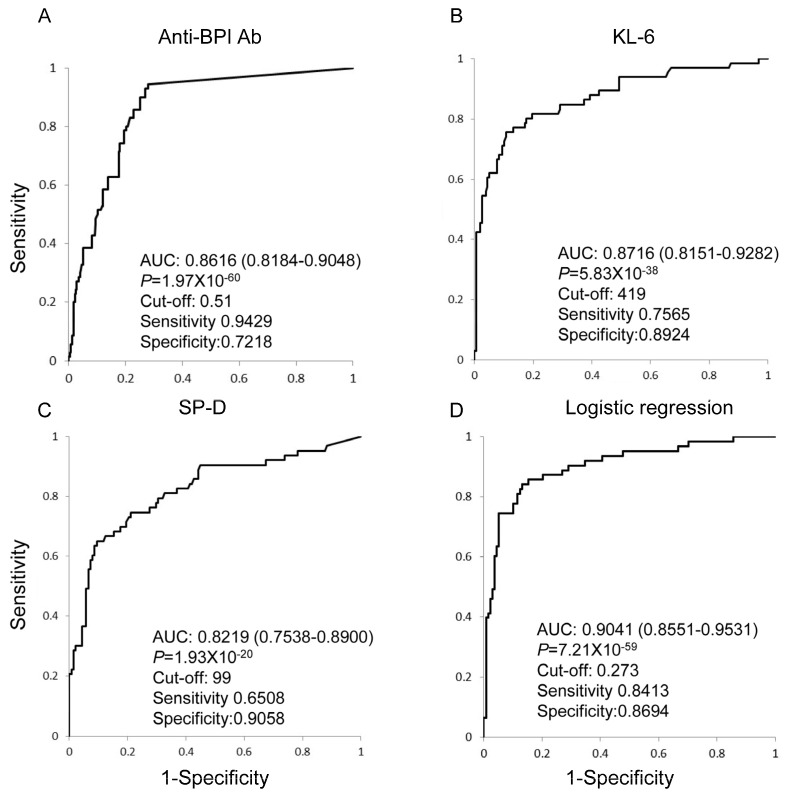
**ROC curves using anti-BPI Abs, KL-6, and SP-D for comparisons between RA+UIP and those without CLD.** ROC curves for anti-BPI Abs (**A**), KL-6 (**B**), SP-D (**C**), and the complex marker generated by logistic regression analyses are shown (**D**). The AUC values of the ROC curves with 95% confidence intervals and the optimized cut-off levels with specificities and sensitivities are described. RA: rheumatoid arthritis, UIP: usual interstitial pneumonia, BPI: bactericidal permeability increasing protein, Ab: antibody, ROC: receiver operating characteristic, AUC: area under the curve, CLD: chronic lung disease, KL-6: Krebs von den lungen-6, SP-D: surfactant protein-D.

Anti-BPI Ab ROC curves were developed to assess differences in RA+UIP and RA−CLD (Figure 2A). AUC values of ROC curves for anti-BPI Abs (0.8616, 95% CI 0.8184–0.9048) were comparable with those for KL-6 (0.8716, 95% CI 0.8151–0.9282, *p* = 0.5933, Figure 2B), or SP-D (0.8219, 95% CI 0.7538–0.8900, *p* = 0.4185, Figure 2C). The sensitivity of anti-BPI Ab was higher than that of KL-6 or SP-D. Multiple logistic regression analyses of anti-BPI Ab, KL-6, and SP-D were performed to generate a complex marker. The AUC value of ROC curves for the complex marker was comparable with that of anti-BPI Abs (0.9041, 95% CI 0.8551–0.9531, *p* = 0.1208, Figure 2D). Thus, anti-BPI Ab values have related properties to KL-6 or SP-D when making a diagnosis of UIP in RA.

**Figure 3:**
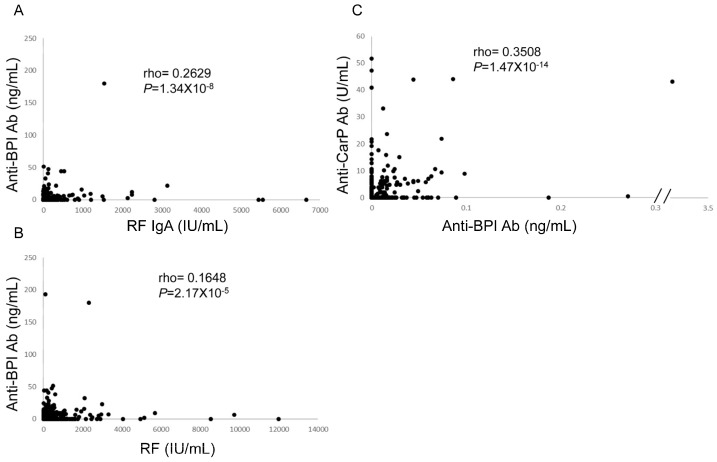
**Correlations of anti-BPI Abs with RF IgA, RF, or anti-CarP Abs.** Spearman correlations between anti-BPI Abs and RF IgA (**A**), anti-BPI Abs and RF (**B**), and anti-BPI Abs and anti-CarP Abs (**C**) are shown. RA: rheumatoid arthritis, BPI: bactericidal permeability increasing protein, Ab: antibody, RF: rheumatoid factor, CarP: carbamylated proteins.

Correlations of anti-BPI Ab titers and other autoantibodies are shown in Figure 3. The association of anti-BPI Ab and RF IgA amounts was determined for RA patients (rank correlation coefficient rho 0.2629, *p* = 1.34 × 10^−8^, Figure 3A). The weaker correlation of anti-BPI Ab amounts and RF levels was also noted in RA patients (0.1648, *p* = 2.17 × 10^−5^, Figure 3B). A stronger correlation of anti-BPI Ab amounts and anti-CarP Ab levels was observed in RA patients (0.3508, *p* = 1.47 × 10^−14^, Figure 3C in different scales). Thus, correlation analysis using anti-BPI Ab and certain autoantibodies showed the co-production of autoantibodies, including anti-BPI Ab.

### 3.2. Anti-BPI Abs in RA Subsets and HCs

Increased anti-BPI Ab levels were noted in RA+UIP (*p* = 0.0017) when differences to HCs were assessed (Table 3). These were also larger in RA with ILD (*p* = 0.0003) and CLD (*p* = 0.0002), as well as overall RA (3.0 ± 11.9 [ng/mL] vs. 1.1 ± 1.4, *p* = 9.67 × 10^−5^). Thus, increased anti-BPI Ab levels were found for overall RA cases, especially in RA+UIP, compared with HCs.

## 4. Discussion

We showed that anti-BPI Abs were linked to UIP in RA, in contrast to a previous study reporting a link between anti-BPI Abs and AD in RA [23]. Another study reported anti-BPI Abs promoted neutrophil extracellular trap formation [32]. These data suggested anti-BPI Abs may be linked to the pathogenesis of ANCA-associated vasculitis, frequently complicated with ILD [33]. Common mechanisms for ILD in RA and ILD in ANCA-associated vasculitis were predicted [34]. Anti-BPI Abs react with neutrophils and cause the production of reactive oxygen species and the release of neutrophil extracellular traps (NETs) through programmed cell death, NETosis. NETs contain proinflammatory mediators and contribute to inflammation. Persistent production of anti-BPI Abs might cause chronic inflammation in the lung, because inflammation of small vessel vasculitis is diffusive to the interalveolar septum. The implications of ANCA and NETs in the pathogenesis of ILD have been suggested; however, the precise pathogeneses are still unknown.

A link between RF, anti-CarP Ab, and RF IgA, with ILD in RA, was previously reported [10,11,13]. We report that anti-BPI Abs are also linked to ILD in RA. A correlation between anti-BPI Ab and anti-CarP Ab levels in RA was also found. These data suggested the co-production of some autoantibodies, including anti-BPI Abs, in the same individuals, suggesting the involvement of common mechanisms in the lung related to the production of some autoantibodies. Anti-*P. gingivalis* Ab levels in RA with CLD were lower compared with RA without CLD. Citrullinated peptides were mainly generated in the lung of RA with CLD, the oral cavity of RA without CLD, and anti-citrullinated peptide Abs would be generated in the lung of RA with CLD or the oral cavity of RA without CLD [35]. These data supported our hypothesis on the involvement of common mechanisms in the lung for the production of anti-BPI Ab and anti-CarP Ab.

AUC values of ROC curves related to KL-6, SP-D, or anti-BPI Abs were akin when RA+UIP and RA−CLD were compared. Since KL-6 and SP-D for ILD in RA were validated in many studies [7,8,36,37], anti-BPI Abs might be a good candidate biomarker for UIP in RA. However, distinctions in the levels of anti-BPI Abs among RA+UIP and RA−CLD were not large. Thus, studies to validate anti-BPI Abs in RA+UIP should be performed in the future. Because radiological exposure could not be ignored during the examination of chest computed tomography and the sensitivity of anti-BPI Ab testing was higher than KL-6 or SP-D, anti-BPI Ab testing would be useful to screen RA patients to be examined for chest computed tomography.

This study showed that anti-BPI Ab amounts in RA were higher than in HCs, in accord with prior reports [17,20]. Because anti-BPI Abs are associated with infection [16,18,22,38], these data might explain the higher incidence rates of infectious diseases in RA patients.

## 5. Conclusions

This study described a link between anti-BPI Ab and UIP in RA. Because the number of patients was modest and it was only conducted in Japanese, validation of our results requires larger-scale multi-ethnic investigations of anti-BPI Abs in RA. In addition, future studies should evaluate anti-BPI Abs in collagen vascular diseases in addition to RA. If anti-BPI Ab could be used for the prediction of prognosis, severity, development or progression, or treatment-response of ILD in RA, it will be clinically useful. Thus, future longitudinal studies on the prediction of prognosis, severity, development or progression, or drug efficacy of ILD in RA should be conducted.

## Figures and Tables

**Table 1:** Anti-BPI Ab in RA patients.

	n	Anti-BPI Ab, ng/mL (SD)	*p*-Value
ILD	167	7.2 (20.9)	0.0004
UIP	70	10.4 (23.5)	0.0020
NSIP	97	5.0 (18.6)	0.0616
AD	167	1.8 (6.5)	0.4403
Emphysema	39	2.0 (7.6)	0.6150
CLD(+)	373	4.2 (15.1)	0.0004
CLD(−)	284	1.3 (3.8)	

RA: rheumatoid arthritis, BPI: bactericidal permeability increasing protein, ILD: interstitial lung disease, UIP: usual interstitial pneumonia, NSIP: nonspecific interstitial pneumonia, AD: airway disease, CLD: chronic lung disease, Ab: antibody. ILD group includes UIP and NSIP groups. CLD(+) group includes UIP, NSIP, AD, and emphysema groups. Average value of each group was shown. Standard deviations are shown in parentheses. Differences were evaluated for comparisons with the CLD(−) population by *t*-test.

**Table 2:** Anti-BPI Ab positivity in RA patients.

	Anti-BPI Ab Positive, n (%)	*p*-Value
ILD	73 (43.7)	3.23 × 10^−11^
UIP	44 (62.9)	4.63 × 10^−15^
NSIP	29 (29.9)	0.0015
AD	17 (10.2)	0.1934
Emphysema	3 (7.7)	0.3247
CLD(+)	93 (24.9)	0.0017
CLD(−)	42 (14.8)	

RA: rheumatoid arthritis, BPI: bactericidal permeability increasing protein, ILD: interstitial lung disease, UIP: usual interstitial pneumonia, NSIP: nonspecific interstitial pneumonia, AD: airway disease, CLD: chronic lung disease, Ab: antibody. ILD group includes UIP and NSIP groups. CLD(+) group includes UIP, NSIP, AD, and emphysema groups. The positive number of anti-BPI Ab in each group was shown. Positive frequencies are shown in parentheses. Differences were evaluated for comparisons with the CLD(−) population by Fisher’s exact test using 2 × 2 contingency tables.

**Table 3:** Comparison of anti-BPI Abs in RA patients and controls.

	*p*-Value
ILD	0.0003
UIP	0.0017
NSIP	0.0513
AD	0.2864
Emphysema	0.5324
CLD(+)	0.0002
CLD(−)	0.1072
RA	9.76 × 10^−5^
	Anti-BPI Ab, ng/mL (SD)
RA (n = 657)	3.0 (11.9)
Controls (n = 52)	1.1 (1.4)

RA: rheumatoid arthritis, BPI: bactericidal permeability increasing protein, ILD: interstitial lung disease, UIP: usual interstitial pneumonia, NSIP: nonspecific interstitial pneumonia, AD: airway disease, CLD: chronic lung disease, Ab: antibody. ILD group includes UIP and NSIP groups. CLD(+) group includes UIP, NSIP, AD, and emphysema groups. Average value of each group was shown. Standard deviations are shown in parentheses. Differences were evaluated for comparisons with the control population by *t*-test.

## Data Availability

Data supporting study results are given in this article and Supplementary Data. Other data can be provided by the authors upon request. Data related to clinical and genotype information for each study individual is not available under the conditions of informed consent mandated by the Act on the Protection of Personal Information.

## Supplementary Materials

The following supporting information can be downloaded at: https://www.mdpi.com/article/10.3390/jcm15145433/s1, Table S1: clinical manifestations of patients with RA. Table S2. Multiple logistic regression analysis of Anti-BPI Ab and clinical manifestations for UIP in RA. Table S3. Multiple logistic regression analysis of Anti-BPI Ab, KL-6, and SP-D for UIP in RA.

## Abbreviations

The following abbreviations are used in this manuscript:

Ab	antibody
AD	airway disease
ANCA	anti-neutrophil cytoplasmic Ab
AUC	area under curve
BPI	bactericidal permeability-increasing protein
CarP	carbamylated protein
CI	confidence interval
CLD	chronic lung disease
ELISA	enzyme-linked immunosorbent assay
HCs	healthy control
Ig	immunoglobulin
ILD	interstitial lung disease
KL-6	Krebs von den lungen-6
NSIP	nonspecific interstitial pneumonia
RA	rheumatoid arthritis
RF	rheumatoid factor
ROC	receiver operating characteristic
SP-D	surfactant protein-D
UIP	usual interstitial pneumonia
