# Supplementary material for: Augmented Anti-Bactericidal Permeability-Increasing Protein Antibody Levels in Rheumatoid Arthritis Patients Complicated by Usual Interstitial Pneumonia
Source: J Clin Med. 2026 Jul 10;15(14):5433. doi: 10.3390/jcm15145433 (PMC13412791; doi:10.3390/jcm15145433)
Supplement: Supplementary file 1 [file jcm-15-05433-s001.zip › jcm-4399022-supplementary.pdf]

Supplementary Table S1. Clinical manifestations of patients with RA.

Clinical manifestations	UIP (n=70)	NSIP (n=97)	AD (n=167)	Emphysema (n=39)	CLD (-) (n=284)
Age, years (SD)	70.0 (10.0)	68.2 (8.0)	67.1 (9.1)	70.9 (9.4)	65.6 (9.4)
Male n (%)	23 (36.5)	14 (18.7)	28 (25.0)	13 (35.1)	49 (27.4)
Age at onset, years (SD)	58.0 (15.7)	54.9 (13.1)	51.1 (15.4)	63.4 (11.7)	52.6 (12.4)
Steinbrocker stage 3, 4, n (%)	29 (47.5)	30 (40.0)	64 (58.2)	10 (29.4)	89 (49.7)
Smoker or past smoker, n (%)	28 (47.5)	24 (34.3)	50 (46.3)	12 (36.4)	65 (39.6)
KL-6, U/mL (SD)	904.7 (849.2)	714.1 (607.7)	504.4 (522.0)	533.3 (473.5)	338.0 (325.0)
SP-D, ng/mL (SD)	149.6 (105.5)	119.2 (164.8)	83.3 (101.8)	101.4 (72.4)	55.9 (43.6)
BMI, kg/m2 (SD)	22.6 (3.3)	23.5 (4.8)	21.0 (3.0)	21.1 (3.8)	21.6 (3.4)
RF, U/mL (SD)	454.9 (888.5)	541.2 (1313.4)	366.4 (899.5)	285.6 (527.3)	357.4 (1073.9)
RF IgA, U/mL (SD)	263.5 (502.0)	170.8 (315.6)	191.5 (841.5)	621.0 (1124.3)	127.7 (567.9)
Anti-CarPAb, U/mL (SD)	0.012 (0.016)	0.064 (0.370)	0.005 (0.013)	0.006 (0.012)	0.004 (0.017)

RA: rheumatoid arthritis, UIP: usual interstitial pneumonia, NSIP: nonspecific interstitial pneumonia, AD: airway disease, CLD: chronic lung disease, KL-6: Krebs von den Lungen-6, SP-D: Surfactant Protein-D, RF: Rheumatoid factor, BMI: Body Mass Index, CarP: carbamylated proteins. Data are presented as the mean value or number of each group. Phenotype frequencies or standard deviations were shown in parenthesis.

Supplementary Table S2. Multiple logistic regression analysis of Anti-BPI Ab and clinical manifestations for UIP in RA.

	Unconditioned			Conditioned on the other factors		
	OR	95%CI	*P*	$OR_{adjusted}$	95%CI	$P_{adjusted}$
Anti-BPI Ab, ng/mL	1.20	(1.13-1.28)	$1.26X10^{-8}$	1.15	(1.08-1.23)	$2.07X10^{-5}$
Age, years	1.09	(1.06-1.13)	$7.37X10^{-8}$	1.09	(1.05-1.13)	$1.11X10^{-5}$
Male	2.77	(1.56-4.90)	0.0005	3.00	(1.38-6.50)	0.0055
Smoker or past smoker	1.77	(1.02-3.07)	0.0440	1.18	(0.57-2.46)	0.6591

RA: rheumatoid arthritis, UIP: usual interstitial pneumonia, OR: odds ratio, CI: confidence interval. *P* , OR, 95%CI, $P_{adjusted}$, $OR_{adjusted}$ were calculated by logistic regression analysis on RA patients.

Supplementary Table S3. Multiple logistic regression analysis of Anti-BPI Ab, KL-6, and SP-D for UIP in RA.

	Unconditioned			Conditioned on the other factors		
	OR	95%CI	*P*	$OR_{adjusted}$	95%CI	$P_{adjusted}$
Anti-BPI Ab, ng/mL	1.20	(1.13-1.28)	$1.26X10^{-8}$	1.10	(1.04-1.16)	0.0009
KL-6, U/mL	1.01	(1.00-1.01)	$7.30X10^{-9}$	1.00	(1.00-1.00)	0.0010
SP-D, ng/mL	1.02	(1.01-1.03)	$1.24X10^{-9}$	1.01	(1.01-1.02)	0.0013

RA: rheumatoid arthritis, UIP: usual interstitial pneumonia, OR: odds ratio, CI: confidence interval. *P* , OR, 95%CI, $P_{adjusted}$, $OR_{adjusted}$ were calculated by logistic regression analysis on RA patients.
